# Syphilitic Interstitial Gingivitis

**Published:** 1901-02

**Authors:** Eugene S. Talbot


### SYPHILITIC INTERSTITIAL GINGIVITIS.

BY EUGENE S. TALBOT, M.D., D.D.S.

A FIFTY-SIX-YEAR-OLD woman was sent to me, November 16, by her family physician. I found her suffering intense pain throughout the superior alveolar process. The teeth of the upper jaw were all in place. Absorption of the alveolar process was marked and extended back from the cuspids upon both sides. The bifurcations of the roots of the molars were exposed. The alveolar process was quite thin over the roots. It had obviously been absorbed laterally as well as in line with the roots. Intense metabolic changes had clearly taken place at some earlier period. The gums and mucous membrane over the anterior alveolar process had a bright-red appearance. The gums were smooth, neither puffy nor engorged with blood, nor did they bleed upon touch. Teeth felt uncomfortable on occlusion with those on the lower jaw. Several of the inferior teeth had been lost. They had become loose and had been easily extracted some years previously. There was no pyorrhœa. Her hair had dropped out some years previously, and was at the time of the examination very scanty. She had had joint-rheumatism for some years. She was neurasthenic. There was profuse leucorrhœa. Application of iodine to the gums produced most intense pain. On consultation with her physician, we decided she was suffering with secondary syphilis. She was given potassium iodide and ordered to drink large quantities of water and to take ten grains of lithia twice daily. She began to improve from the beginning of the treatment in a marked manner. The pain ceased and the tissues are now nearly normal.

